# Modeling Cytomegalovirus Infection in Mouse Tumor Models

**DOI:** 10.3389/fonc.2015.00061

**Published:** 2015-03-17

**Authors:** Richard Lee Price, Ennio Antonio Chiocca

**Affiliations:** ^1^Department of Neurological Surgery, Washington University, St. Louis, MO, USA; ^2^Harvey Cushing Neuro-Oncology Laboratories, Harvard Institutes of Medicine, Department of Neurosurgery and Institute for the Neurosciences, Brigham and Women’s Faulkner Hospital and Center for Neuro-Oncology, Dana-Farber Cancer Institute, Boston, MA, USA

**Keywords:** cytomegalovirus, malignant glioma, glioblastoma, rhabdomyosarcoma, mouse model, oncomodulatory

## Abstract

The hypothesis that cytomegalovirus (CMV) modulates cancer is evolving. Originally discovered in glioblastoma in 2002, the number of cancers, where intratumoral CMV antigen is detected, has increased in recent years suggesting that CMV actively affects the pathobiology of certain tumors. These findings are controversial as several groups have also reported inability to replicate these results. Regardless, several clinical trials for glioblastoma are underway or have been completed that target intratumoral CMV with anti-viral drugs or immunotherapy. Therefore, a better understanding of the possible pathobiology of CMV in cancer needs to be ascertained. We have developed genetic, syngeneic, and orthotopic malignant glioma mouse models to study the role of CMV in cancer development and progression. These models recapitulate for the most part intratumoral CMV expression as seen in human tumors. Additionally, we discovered that CMV infection in Trp53^−/+^ mice promotes pleomorphic rhabdomyosarcomas. These mouse models are not only a vehicle for studying pathobiology of the viral-tumor interaction but also a platform for developing and testing cancer therapeutics.

## Introduction

Mounting evidence suggest a role for persistent human cytomegalovirus (HCMV) infection in multiple types of cancer (1). The hypothesis was originally suggested in 2002 by a paper reporting detection of HCMV-specific gene expression by immunohistochemistry (IHC) for multiple viral proteins (HCMV-IE1 and HCMV-pp65) and *in situ* hybridization (ISH) for HCMV DNA in 100% of glioblastomas and astrocytomas tested (*n* = 22) (2). Viral antigens were not present in normal brain regions from the same patients or in other brain abnormalities from other patients. Using similar sensitive techniques, other groups were able to detect HCMV in tumors (3, 4). Additionally, HCMV infection directly correlated with the grade of glioma. Higher-grade tumors exhibited increased HCMV immune-positive cells in the tumor. There was also increased HCMV immune-positivity associated with higher tumor grade (4). Recent studies have provided additional evidence that HCMV exists in malignant glioma as well as provided more insight into how this virus modulates the cancer. Besides HCMV-IE1 and -pp65, two studies additionally found HCMV-US28 in glioblastomas (5, 6). HCMV-US28 is an oncomodulatory protein that is capable of activating signal transducer and activator of transcription 3 (STAT3) (5). Other groups have focused on detecting the HCMV genome in glioblastoma. One study detected 20 different HCMV genes in most glioblastoma cases tested (7). The most comprehensive study sequenced HCMV in glioblastomas after detecting the virus in 94% of tumors (8). They were able to successfully sequence part of the HCMV genome. Interestingly, the tumor-associated sequences showed significant variability with published HCMV genomes. Another study correlated HCMV infection with glioblastoma survival (9). They discovered that low-grade HCMV infection (<25% HCMV positive cells) was associated with long-term survival in glioblastoma patients (>18 months). These data suggest that high-viral load within the tumor significantly decreases survival. However, it should be noted that this topic remains highly controversial. In fact, several recent papers continue to refute the finding that HCMV is present in tumors. One group used next generation sequencing to detect possible viral DNA sequences in 21 malignant gliomas (10), but failed to detect HCMV DNA. Another group similarly could not detect HCMV DNA in 34 glioblastoma cases utilizing deep-coverage whole-genome sequencing (11). These negative findings continue to render the role of CMV in cancer highly controversial.

Besides malignant gliomas, HCMV protein has also been detected in additional cancers: prostate, mucoepidermoid carcinoma, lung carcinoma, colorectal cancer, and breast cancer (12–16), and we have demonstrated the presence of CMV in rhabdomyosarcomas (RMS) (17). Further, HCMV was detected in 92% of medulloblastomas in one study (18). In this particular report, to validate their finding *in vivo*, the authors treated human medulloblastoma flank tumors with ganciclovir, celecoxib, or a combination of both. Drug therapy shrunk the tumors suggesting that HCMV presence in tumors may be a target for drug therapy (19). The authors of one clinical trial concluded that Valcyte is effective in extending the survival of patients with CMV^+^ glioblastomas (20), although this conclusion has been disputed by others, based on limitations of the trial design and conduct (21). One recent report from a phase 1 trial in Australia reported encouraging data related to safety and possible effectiveness for a CMV-targeted adoptive T cell therapy (22). In fact, anti-CMV immunotherapy is now being tested in multiple human trials for patients with recurrent glioblastoma. Therefore, in spite of the controversy, clinical trials targeting CMV in tumors are proceeding, thus rendering our need to understand the role of this tumor pathobiology even more pressing.

In summary, although a strong association between CMV and multiple cancer types has been suggested by the aforementioned studies, they fall short of proving a causal relationship or unequivocally explaining the effect of HCMV infection on cancer pathobiology. High rates of HCMV seropositivity render epidemiological linkage of HCMV with uncommon diseases, such as malignant glioma, highly difficult to demonstrate. In addition, there is no evidence that CMV is related to or modulates cancer in an *in vivo* setting. Another possibility may be that HCMV, while not causative on its own, could still be a modulating factor in cancer pathobiology (23). Our group has developed mouse models to try and study the role of CMV infection in cancer progression. To show that CMV can function as a cancer “modulator,” we employed genetically engineered mouse models. Based on the particular context of tumor suppressor mutations in the mice, we have discovered a link between CMV and malignant gliomas as well as RMS. In this review, we plan to provide details of the animal methodologies employed as well as summarize our most salient findings and discuss the need for additional studies.

## Viral Infection Methodology

To test the hypothesis that CMV affects cancer development we combined *in vivo* cancer mouse models with MCMV infection protocols. Cytomegaloviruses (CMVs) are strictly species-specific (24, 25) and several different CMV types have been identified for many mammals (i.e., human, mouse, guinea pig, etc.). MCMV and HCMV are similar in size and virion structure. However, their genomic sequences are not identical. Despite the difference in genome sequence, MCMV is functionally homologous to HCMV. The viruses share the same properties with respect to genome structure (not sequence), pattern of gene expression, cell tropism, and infectious dynamics (26–29). Therefore, MCMV is a generally accepted model for HCMV infection, latency, reactivation, and pathogenesis. A combination of mouse genetic and orthotopic models were developed in order to rigorously test the complex role of CMV in the tumors. To study the role of CMV in malignant gliomas, initially we utilized the Mut3 mouse model that spontaneously develops high-grade astrocytomas (WHO grade III anaplastic astrocytoma and grade IV glioblastoma) with almost complete penetrance by adulthood (30). These mice developed normally until they became symptomatic for glioma (i.e., seizures, weight loss, lethargy, paralysis) as adults.

Direct intracerebral inoculation is an efficient model of neurological infection, but these mice eventually succumb to viral encephalitis (31). Unfortunately, this prohibits study of chronic diseases such as cancer. Koontz and colleagues developed an infection protocol using i.p. injection to study MCMV infection in the brain (32). This model delivers MCMV via i.p. injection, leading to systemic infection, including brain infection. In this model, MCMV was detected in the brain at an early age, which proved to be an ideal method to studying the role of CMV in gliomas as it closely mimics human infection patterns.

Mut3 (*GFAP-cre*; *Nf1*^loxP/+^; *Trp53*^−/+^) male mice were bred with wild type (wt) B6CBAF1/J females to generate mice of desired genotypes (30). They were bred in a CMV-free animal vivarium. Introducing an infectious pathogen into a vivarium is a risky endeavor. Without careful monitoring and strict infection protocols, unintended spread of infection can occur. We collaborated extensively with veterinarians and animal facility staff to develop protocols to allow us to pursue experiments without unintentionally spreading infection. Pregnant females were transferred to an isolated vivarium that was specifically designed to hold only infected mice, thus reducing the risk of infectious spread to the general non-infected mouse colony. To prevent viral spread, MCMV and mock-infected mice were housed on separate mouse cage racks, mock-infected mice were handled before infected mice, and cages were changed on different days. Mice were intraperitoneally (i.p.) injected on postnatal day 2 (P2) mice with 10^3^ plaque forming units (p.f.u.s.) of MCMV-m157 in 100 μl of phosphate-buffered saline (PBS) or PBS only (mock). The lack of the *m157* gene increases virulence in B6 mice (33). Another cohort of mice received 10^3^ p.f.u.s. of a different neurotropic virus, HSV1 F strain (34), via i.p. injection in 100 μl of PBS as a viral control.

Mice infected with MCMV were behaviorally indistinguishable from mock-infected mice. Mice were sacrificed at desired time points and were perfused via an intracardiac route with PBS. Mice for IHC were additionally perfused with 4% paraformaldehyde. To verify infection, we sacrificed P2 mock- or MCMV-infected mice at 8 weeks of age. Mice were perfused with PBS and tissue of interest was dissected out and total DNA was purified.

## Experimental Results of Cytomegalovirus Infection in Malignant Glioma Mouse Models

**Figure 1:**
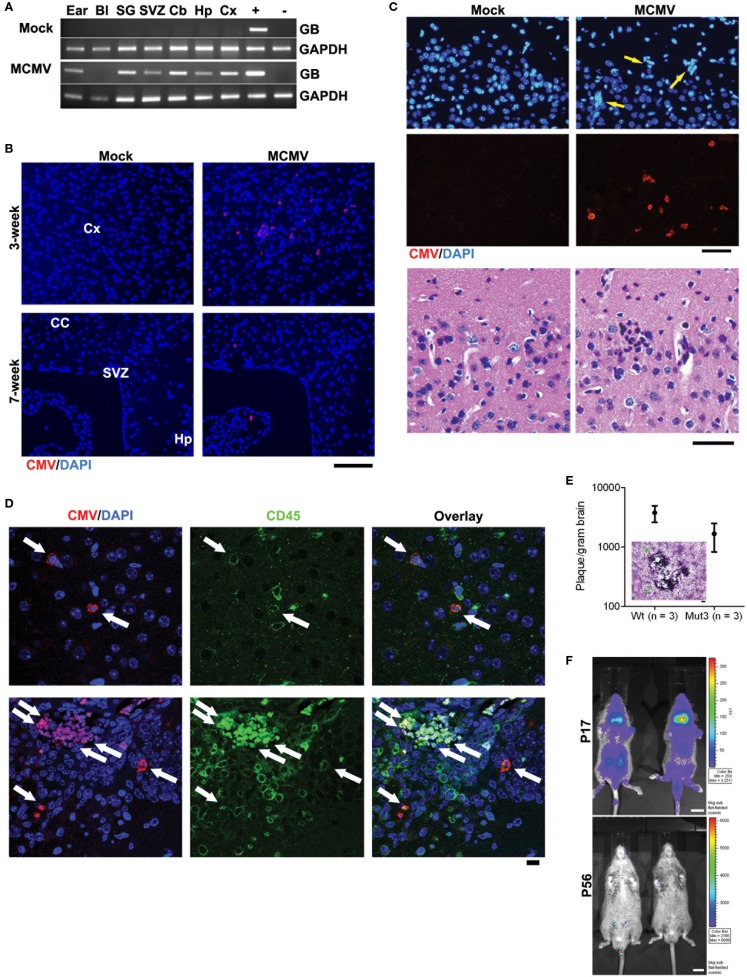
**Perinatal MCMV infection resulted in productive virus replication and CMV immunoreactivity in CD45-positive cells in the brain**. **(A)** PCR analysis for MCMV glycoprotein B (GB) gene in genomic DNA obtained from PBS-perfused mock- versus MCMV-infected, 8-week-old mice. Bl, blood; SG, salivary gland; Cb, cerebellum; Hp, hippocampus; Cx, cortex. “+” and “−”: gDNA from MCMV-infected and -negative cells, respectively. **(B)** IHC data for CMV in the brains of mock- versus MCMV-infected mice. Cx, cortex; CC, corpus callosum; Hp, hippocampus. Scale bar = 100 mm. **(C)** Top, IHC images show ectopic cellularity (blue, arrows) close to CMV-positive cells (red) in the cortex of 3-week-old wt mice infected with MCMV at P2, which were absent in mock-infected mice. Bottom, hematoxylin and eosin (H&E)-stained brain sections adjacent to the sections in top images contained an inflammatory focus (arrow) in the MCMV mice, which was absent in mock-infected mice. **(D)** Confocal images of IHC for CMV and CD45 display immunoreactivity in the brains from 2-week-old wt mice. Cells double positive for both CMV and CD45 are pointed by arrows. Scale bar = 10 mm. **(E)** Brain extract of 2-week-old mice infected with MCMV formed virus-induced plaques in 3T3 cells. There was no significant difference between wt and Mut3 (*P* = 0.16). Error bars indicate SEM. Inset, a representative image of plaque formation (arrows) in 3T3 cells. Scale bar = 100 mm. No plaque was formed by brain extract of mock-infected mice. **(F)** Bioluminescent live imaging on P17 and adult (P56) mice infected with MCMV-luciferase. Scale bar = 1 cm. Reprinted by permission from the American Association for Cancer Research: Price et al. (38).

Using the aforementioned model, MCMV-GB gene was consistently detected by polymerase chain reaction (PCR) in multiple tissues, including the brain, from MCMV- but not from mock-infected (Figure 1A). This similar pattern has been demonstrated by other groups and is a reliable marker of systemic infection (35). At 3 weeks of age, diffuse MCMV protein was evident in the brain from MCMV-, but not mock-infected mice (Figure 1B). Additionally, regions of immunoreactivity demonstrated microgrial nodules as seen on H&E staining (Figure 1C, bottom), which is consistent with data from Koontz and colleagues (32). This suggests that MCMV infects Mut3 mice similarly to wt mice. Additionally, MCMV reaches the brain and causes an immune reaction as evidenced by the microglial nodules. By 7 weeks of age, MCMV-reactive cells decreased and were mostly restricted to specific brain regions, including the hippocampus and subventricular zone (Figure 1C). Reduction of MCMV antigen expression is presumably due to immune clearance of virus and resulting latency. Interestingly, the hippocampus and subventricular zone are rich in neural stem cells, a cell type demonstrated to give rise to malignant gliomas (36). We performed dual antigen IHC on cell markers for all neural cell types to determine what cell type was infected. MCMV did not co-stain with neural markers, but did co-localize with CD45^+^ cells (Figure 1D). This data suggest that CMV is found in lymphocytes in the brain as opposed to native neural cells. Data from intracerebral infection models demonstrate that MCMV infects a variety of neural cell types depending on viral phase (29). The i.p. infection model suggests that MCMV traffics to the brain via CD45^+^ immune cells. Whether this is true in humans is yet to be determined. To differentiate whether live virus was in brain, and that we were not just detecting viral antigens, whole brain extracts from 2-week-old MCMV-infected mice were plated over a monolayer of fibroblasts. Both Mut3 and wt mice formed infectious plaques when plated on fibroblasts *in vitro* (Figure 1E), indicating the presence of active MCMV replication. There was no statistical difference in viral activity between Mut3 and wt mice suggesting that MCMV infects both genotypes of mice equally. Another cohort of mice was infected with a luciferase-tagged MCMV (MCMV-luc) to study the systemic spread of infection. After infection, the mice were serially imaged to test for bioluminescence. Systemic infection peaked at day 17 and was undetectable by day 56 (Figure 1F). This infection pattern is consistent with patterns seen in humans: active infection after inoculation followed by latency after the virus is cleared by the immune system (37).

**Figure 2:**
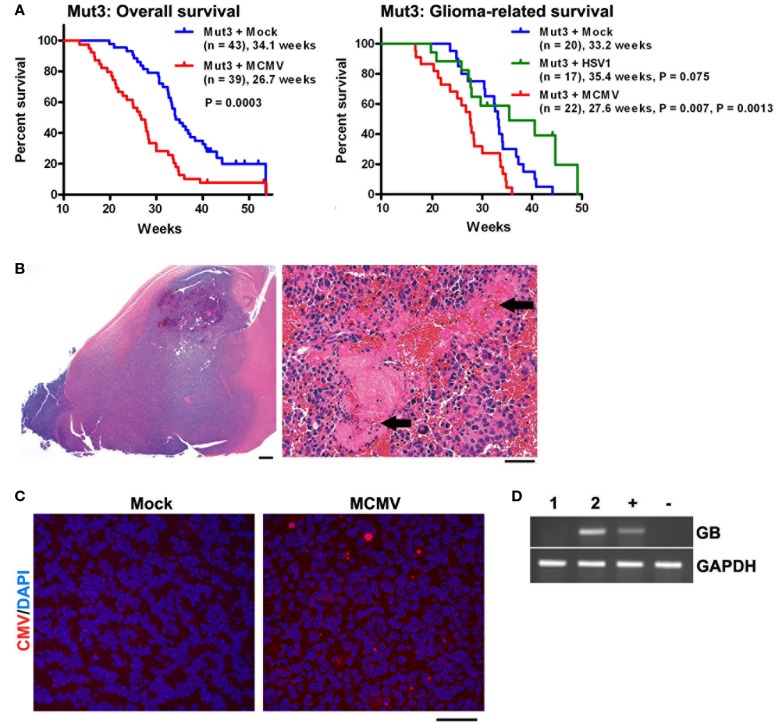
**Perinatal MCMV infection shortens the survival of Mut3 mice**. **(A)** Kaplan–Meier curves display overall (left) and histologically glioma-confirmed (right) survivals of Mut3 mice. Labels indicate infection groups, mouse numbers, mean survival time, and *P* values against mock and HSV1 (first and second *P* values, respectively). **(B)** Left, a representative hematoxylin and eosin (H&E)-stained brain section from an MCMV-infected Mut3 mouse that harbors a grade IV astrocytoma (glioblastoma) spreading extensively throughout the forebrain. Scale bar = 500 mm. Right, a higher magnification image from the same tumor displaying extensive necrosis (arrows). Scale bar = 50 mm. **(C)** IHC images for CMV in gliomas from mock- versus MCMV-infected Mut3 mice. Scale bar = 50 mm. **(D)** RT-PCR analysis for MCMV glycoprotein B (GB) in gliomas from mock- (i) versus MCMV-infected (ii) Mut3 mice. “+” and “−”: MCMV-infected and -negative cells, respectively. Reprinted by permission from the American Association for Cancer Research: Price et al. (38).

After infection was confirmed, Mut3 mice were monitored until they developed signs of brain tumor burden (seizures, lethargy, failure to thrive, etc.). MCMV-infected Mut3 mice died significantly earlier than mock-infected Mut3 mice both as a group and in the subset of mice in which glioma formation was confirmed histologically (Figure 2A). Survival of mock-infected mice was similar to published reports (30). The mean survival of Mut3 mice was decreased by 7.4 weeks in the MCMV group compared to mock-infected mice, corresponding to an approximately 22% shortened life span. MCMV-infected wt mice did not develop brain tumors nor exhibit a shortened life span relative to mock-infected wt mice. Infection of Mut3 mice with HSV1, a different neurotropic virus, did not significantly change life span caused by malignant gliomas when compared to controls (Figure 2B). This suggested that the shortened life span in the context of Mut3 mutations was specific to perinatal MCMV infection rather than a non-specific effect from perinatal infection with any neurotropic virus. This is the first published evidence that CMV can impact malignant gliomas using an *in vivo* model. Symptomatic mice developed gliomas (Figure 2B), some characterized as grade IV glioblastomas with histological evidence of necrosis (Figure 2B, right panel). Malignant gliomas from MCMV-infected mice demonstrated intratumoral MCMV protein expression (Figure 2C) and were positive for MCMV gene expression, unlike control tumors from mock-infected Mut3 mice (Figure 2D). The expression of intratumoral CMV is consistent with data from human studies (2, 4). This suggests that this model replicates the human condition for the most part. In MCMV-infected mice, MCMV expression occurs at high frequency intratumorally. Published data demonstrate a higher number of CMV^+^ cells in human tumors than seen in our murine data. One possible explanation may be that intratumoral immune responses in mice suppress CMV expression more than seen in humans. Additionally, humans typically live longer with malignant gliomas than mice do, months as opposed to days to weeks. Perhaps, this additional time may increase intratumoral CMV expression as seen in humans. The final possibility is that the animal model does not completely replicate the human condition and that MCMV remains strictly lymphocyte tropic while HCMV can infect human glioma cells.

To further validate these findings, we then used a syngeneic, orthotopic malignant glioma model. Intracranial injection of malignant glioma cells cultured from Mut4 mice (*GFAP-cre*; *Nf1*^loxP/+^; *Trp53*^−/+^; *pTen^loxP/^*^+^) (39) into the striatum of wt mice recapitulates glioblastoma phenotypes of necrosis and microvascular proliferation (40). This approach allows for the same tumor burden to be injected into the same brain region, resulting in consistent tumor formation in a pre-defined brain region. This model thus offers some advantages compared to the model in genetically engineered Mut3 mice. It reduces the need for genetic breeding and allows for less variable tumor formation, both temporally and spatially. However, it is an artificial system requiring a major procedure to inject the brain tumor cells. Wt mice were infected with mock or MCMV at P2 were intracranially injected with MCMV-free mouse glioblastoma cells at 8 weeks of age. MCMV-infected wt recipients died significantly earlier than mock-infected mice. Mean survival was decreased by 1.8 weeks, corresponding to an approximately 19% shortened survival similar to data from the Mut3 spontaneous glioma model. The similar reduction in survival suggests that host infection, as opposed to intrinsic tumor infection, leads to a more lethal tumor. This is congruent with earlier data showing that MCMV is active in CD45^+^ lymphocytes. This infected cell type potentially modulates malignant glioma into a more lethal phenotype via paracrine signaling.

Additionally, we created an orthotopic model using athymic mice to study the effect of HCMV on human malignant glioma cells. Patient-derived human GBM neurospheres were infected with HCMV and then dissociated three days later. HCMV- or mock-infected dissociated cells were injected in the flanks of athymic mice and then monitored for survival. HCMV-infected tumors grew larger. These tumors were then removed and analyzed. This technique demonstrates one method of studying the HCMV effect on human tumors using an *in vivo* system. Another group developed a similar model to study medulloblastoma (18). This group showed the presence of HCMV in the tumor and that these tumors could be treated with an anti-CMV agent. Directly comparing the effects of HCMV on human tumors allows a better insight of what is actually occurring in humans as well as allows the development of therapeutics to treat CMV-driven tumors.

## MCMV-Infected *Trp53*^−/+^ Mice Develop Pleomorphic RMS

**Figure 3:**
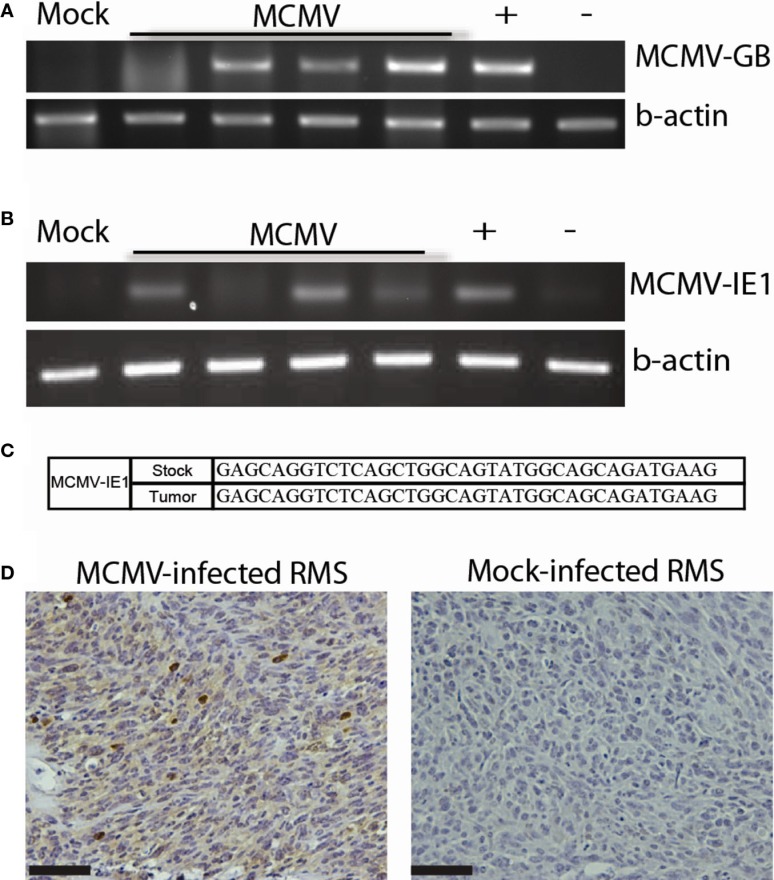
**MCMV analysis of mouse tumors**. **(A)** PCR for MCMV DNA in tumors from MCMV (*n* = 4) and mock-infected (*n* = 1) mice. **(B)** RT-PCR for MCMV-IE1 in tumors from MCMV (*n* = 4) and mock-infected (*n* = 1) mice. **(C)** RT-PCR product fidelity was validated by sequencing the amplified MCMV-IE1. **(D)** MCMV-IE1 immunohistochemistry in MCMV- and Mock-infected tumor. Scale bars = 50 μm. Reprinted by permission from the American Association for Cancer Research: Price et al. (17).

In addition to malignant gliomas, we are interested in studying the role of CMV in other tumors. Many human tumor types are deficient in the p53 tumor suppressor. *Trp53*^−/+^ mice were derived from Mut3 mice. Published data show that *Trp53*^−/+^ mice develop variable tumors at a late age. Tumors from these mice range from lymphomas to fibrosarcomas, and only rarely result in RMS (<10% of the time) (41). We infected these mice neonatally with MCMV, similarly to our malignant glioma studies, or at 4 weeks of age (10^6^ p.f.u.s) to simulate early adult MCMV infection. Since *Trp53*^−/+^ mice start developing tumors at a late age (>9 months), we terminated experiments at 9 months of age to detect MCMV-specific effects on tumor development. We hypothesized that MCMV would lead to an earlier tumor onset. Neonatal MCMV infection was associated with a statistically significant increase in tumor occurrence compared to mock-infected mice. In fact, only 1 mock-infected out of 27 mice developed a tumor during our study. In contrast, 42.8% (12/28) of neonatally MCMV-infected *Trp53*^−/+^ mice developed tumors at significantly earlier time points when compared to mock-infected mice. Interestingly, 84.6% (10/12) of these tumors were pleomorphic RMS. This data suggest that, not only does MCMV accelerate tumor formation in mice with heterozygous *Trp53* mutation but preferentially leads to formation of pleomorphic RMS as opposed to other types of cancer. Mice infected as adults did not show increased tumor incidence indicating that adult infection does not promote tumor formation. Analysis of these tumors demonstrated that MCMV genetic material was present in tumors as was observed in human tumors. MCMV-infected mice revealed the presence of viral DNA (Figure 3A) in all tumors tested, but not in the mock-infected *Trp53*^−/+^ mouse. Additionally, early gene MCMV-IE1 transcripts were present in most tumors, whereas a gene transcribed later in the replication cycle, MCMV-GB, was not detected (Figure 3B and not shown). We do not know why this late gene was not transcribed, but this suggests that full viral replication is not occurring. Instead, the virus is only transcribing certain early transcripts. Sequencing of the transcription product from RT-PCR reactions of MCMV-IE1 RNA revealed that the transcripts sequenced from the tumor matches the stock virus injected into the mice (Figure 3C). Furthermore, MCMV-infected tumors expressed IE1 protein (Figure 3D). Strong intratumoral IHC staining corroborates the human data. Taken together, these data show transcriptionally active MCMV within RMS tumors, which could modulate the tumor. These data suggest a role of CMV in RMS development. Our data show for the first time that CMV infection combined with *Trp53* heterozygosity promotes pleomorphic RMS. Furthermore, the idea that CMV can promote tumorigenesis in an organism with genetic aberrations, such as loss of a tumor suppressor, may help to explain the difficulty of epidemiologically linking CMV infection with relatively rare cancers.

## Conclusion

We have successfully developed multiple mouse models to test the role of CMV in malignant gliomas. The combination of spontaneous, orthotopic, and human glioma models can be used to study the effect of CMV infection on cancer development. Additionally, we infected *Trp53*^−^*^/^*^+^ with MCMV and discovered that these mice develop pleomorphic RMS. Our data demonstrate that we have created relevant, reproducible mouse models to represent the human condition. The insight gained from these models suggests that HCMV has an active role in tumor progression in humans and that more investigation is necessary. Data from our RMS studies emphasized the importance of neonatal infection as opposed to adult infection. Additionally, MCMV expression occurs in CD45 cells in the brain. Oncomodulatory effects occur via paracrine signaling. After tumor formation, there is an appreciable increase in MCMV expression in the tumor, but not in other normal parts of the mouse. This is similar to IHC of human tumors.

The mouse models can be helpful to further study the possible role of CMV infection in cancer. Moving forward the models can be expanded to investigate other aspects of the role that CMV plays in cancer. As we have demonstrated, it can be adapted to study other cancers besides malignant glioma. Other mouse models of cancer may amenable to CMV infection and should be implemented as the increasing number of tumor types that contain CMV are discovered. Additionally, the role of specific CMV proteins may be dissected by creating chimeric mouse models that express CMV proteins in certain tissues. One model expressing HCMV-US28 has already been developed to study colon cancer (42). New mouse models need to be developed with other oncomodulatory proteins. Also, humanized models that are permissive to HCMV and capable of harboring human tumors will powerfully mimic the human condition. Current models have already demonstrated that anti-CMV therapies can curtail growth of CMV-infected tumors. As better models are created in the future, a stronger understanding of the role of HCMV in human cancer will be garnered. Ultimately, these models will hopefully be utilized to develop cancer therapies that exploit CMV for cancer therapeutics.

## Conflict of Interest Statement

Ennio Antonio Chiocca is a consultant for Alcyone Biosciences, Inc. and DNAtrix, Inc. The contents of this manuscript do not involve products from these companies. Richard Lee Price declares that the research was conducted in the absence of any commercial or financial relationships that could be construed as a potential conflict of interest.

## References

[1] Miller G Brain cancer. A viral link to glioblastoma? Science (2009) 323(5910):30–1 10.1126/science.323.5921.1554 19119195

[2] Cobbs CS Harkins L Samanta M Gillespie GY Bharara S King PH Human cytomegalovirus infection and expression in human malignant glioma. Cancer Res (2002) 62(12):3347–50. 12067971

[3] Mitchell DA Xie W Schmittling R Learn C Friedman A McLendon RE Sensitive detection of human cytomegalovirus in tumors and peripheral blood of patients diagnosed with glioblastoma. Neurooncology (2008) 10(1):10–8. 10.1215/15228517-2007-035 17951512 PMC2600830

[4] Scheurer ME Bondy ML Aldape KD Albrecht T El-Zein R. Detection of human cytomegalovirus in different histological types of gliomas. Acta Neuropathol (2008) 116(1):79–86. 10.1007/s00401-008-0359-1 18351367 PMC3001277

[5] Slinger E Maussang D Schreiber A Siderius M Rahbar A Fraile-Ramos A HCMV-encoded chemokine receptor US28 mediates proliferative signaling through the IL-6-STAT3 axis. Sci Signal (2010) 3(133):ra58. 10.1126/scisignal.2001180 20682912

[6] Soroceanu L Matlaf L Bezrookove V Harkins L Martinez R Greene M Human cytomegalovirus US28 found in glioblastoma promotes an invasive and angiogenic phenotype. Cancer Res (2011) 71(21):6643–53. 10.1158/0008-5472.CAN-11-0744 21900396 PMC3206211

[7] Ranganathan P Clark PA Kuo JS Salamat MS Kalejta RF. Significant association of multiple human cytomegalovirus genomic loci with glioblastoma multiforme samples. J Virol (2012) 86(2):854–64. 10.1128/JVI.06097-11 22090104 PMC3255835

[8] Bhattacharjee B Renzette N Kowalik TF. Genetic analysis of cytomegalovirus in malignant gliomas. J Virol (2012) 86(12):6815–24. 10.1128/JVI.00015-12 22496213 PMC3393585

[9] Rahbar A Stragliotto G Orrego A Peredo I Taher C Willems J Low levels of human cytomegalovirus infection in glioblastoma multiforme associates with patient survival; -a case-control study. Herpesviridae (2012) 3:3. 10.1186/2042-4280-3-3 22424569 PMC3348037

[10] Cimino PJ Zhao G Wang D Sehn JK Lewis JS Duncavage EJ. Detection of viral pathogens in high grade gliomas from unmapped next-generation sequencing data. Exp Mol Pathol (2014) 96(3):310–5. 10.1016/j.yexmp.2014.03.010 24704430

[11] Tang K-W Hellstrand K Larsson E. Absence of cytomegalovirus in high-coverage DNA sequencing of human glioblastoma multiforme. Int J Cancer (2014) 136(4):977–81. 10.1002/ijc.29042 24961996

[12] Melnick M Sedghizadeh PP Allen CM Jaskoll T. Human cytomegalovirus and mucoepidermoid carcinoma of salivary glands: cell-specific localization of active viral and oncogenic signaling proteins is confirmatory of a causal relationship. Exp Mol Pathol (2012) 92(1):118–25. 10.1016/j.yexmp.2011.10.011 22101257

[13] Harkins L Volk AL Samanta M Mikolaenko I Britt WJ Bland KI Specific localisation of human cytomegalovirus nucleic acids and proteins in human colorectal cancer. Lancet (2002) 360(9345):1557–63. 10.1016/S0140-6736(02)11524-8 12443594

[14] Samanta M Harkins L Klemm K Britt WJ Cobbs CS. High prevalence of human cytomegalovirus in prostatic intraepithelial neoplasia and prostatic carcinoma. J Urol (2003) 170(3):998–1002. 10.1097/01.ju.0000080263.46164.97 12913758

[15] Giuliani L Jaxmar T Casadio C Gariglio M Manna A D’Antonio D Detection of oncogenic viruses SV40, BKV, JCV, HCMV, HPV and p53 codon 72 polymorphism in lung carcinoma. Lung Cancer (2007) 57(3):273–81. 10.1016/j.lungcan.2007.02.019 17400331

[16] Harkins LE Matlaf LA Soroceanu L Klemm K Britt WJ Wang W Detection of human cytomegalovirus in normal and neoplastic breast epithelium. Herpesviridae (2010) 1(1):8. 10.1186/2042-4280-1-8 21429243 PMC3063230

[17] Price RL Bingmer K Harkins L Iwenofu OH Kwon C-H Cook C Cytomegalovirus infection leads to pleomorphic rhabdomyosarcomas in Trp53± mice. Cancer Res (2012) 72(22):5669–74. 10.1158/0008-5472.CAN-12-2425 23002204 PMC3500419

[18] Baryawno N Rahbar A Wolmer-Solberg N Taher C Odeberg J Darabi A Detection of human cytomegalovirus in medulloblastomas reveals a potential therapeutic target. J Clin Invest (2011) 121(10):4043–55. 10.1172/JCI57147 21946257 PMC3195466

[19] Hadaczek P Ozawa T Soroceanu L Yoshida Y Matlaf L Singer E Cidofovir: a novel antitumor agent for glioblastoma. Clin Cancer Res (2013) 19(23):6473–83. 10.1158/1078-0432.CCR-13-1121 24170543 PMC3919795

[20] Söderberg-Nauclér C Rahbar A Stragliotto G Survival in patients with glioblastoma receiving valganciclovir. N Engl J Med (2013) 369(10):985–6 10.1056/NEJMc1302145 24004141

[21] Wick W Platten M CMV infection and glioma, a highly controversial concept struggling in the clinical arena. Neuro Oncol (2014) 16(3):332–3 10.1093/neuonc/nou174.82 24523454 PMC3922527

[22] Schuessler A Smith C Beagley L Boyle GM Rehan S Matthews K Autologous T-cell therapy for cytomegalovirus as a consolidative treatment for recurrent glioblastoma. Cancer Res (2014) 74(13):3466–76. 10.1158/0008-5472.CAN-14-0296 24795429

[23] Hollon TC Price RL Kwon C-H Chiocca EA. Mutations in glioblastoma oncosuppressive pathways pave the way for oncomodulatory activity of cytomegalovirus. Oncoimmunology (2013) 2(9):e25620. 10.4161/onci.25620 24319635 PMC3850294

[24] Fioretti A Furukawa T Santoli D Plotkin SA. Nonproductive infection of guinea pig cells with human cytomegalovirus. J Virol (1973) 11(6):998–1003. 4351465 10.1128/jvi.11.6.998-1003.1973 PMC355209

[25] Barabas G Wroblewska Z Gilden DH. Growth of murine cytomegalovirus in murine and heterologous brain cell cultures. Brief report. Arch Virol (1980) 65(2):193–200. 10.1007/BF01317331 6252869

[26] Rawlinson WD Farrell HE Barrell BG. Analysis of the complete DNA sequence of murine cytomegalovirus. J Virol (1996) 70(12):8833–49. 8971012 10.1128/jvi.70.12.8833-8849.1996 PMC190980

[27] Reddehase MJ Podlech J Grzimek NKA. Mouse models of cytomegalovirus latency: overview. J Clin Virol (2002) 25(Suppl 2):S23–36. 10.1016/S1386-6532(02)00195-6 12361754

[28] Krmpotic A Bubic I Polic B Lucin P Jonjic S Pathogenesis of murine cytomegalovirus infection. Microbes Infect (2003) 5(13):1263–77 10.1016/j.micinf.2003.09.007 14623023

[29] Tsutsui Y Kosugi I Kawasaki H. Neuropathogenesis in cytomegalovirus infection: indication of the mechanisms using mouse models. Rev Med Virol (2005) 15(5):327–45. 10.1002/rmv.475 16100703

[30] Alcantara Llaguno S Chen J Kwon C-H Jackson EL Li Y Burns DK Malignant astrocytomas originate from neural stem/progenitor cells in a somatic tumor suppressor mouse model. Cancer Cell (2009) 15(1):45–56. 10.1016/j.ccr.2008.12.006 19111880 PMC2650425

[31] Kossmann T Morganti-Kossmann MC Orenstein JM Britt WJ Wahl SM Smith PD. cytomegalovirus production by infected astrocytes correlates with transforming growth factor-beta release. J Infect Dis (2003) 187(4):534–41. 10.1086/373995 12599069

[32] Koontz T Bralic M Tomac J Pernjak-Pugel E Bantug G Jonjic S Altered development of the brain after focal herpesvirus infection of the central nervous system. J Exp Med (2008) 205(2):423–35. 10.1084/jem.20071489 18268036 PMC2271002

[33] Bubić I Wagner M Krmpotić A Saulig T Kim S Yokoyama WM Gain of virulence caused by loss of a gene in murine cytomegalovirus. J Virol (2004) 78(14):7536–44. 10.1128/JVI.78.14.7536-7544.2004 15220428 PMC434107

[34] Ben-Hur T Rosen-Wolff A Lamade W Darai G Becker Y. HSV-1 DNA sequence determining intraperitoneal pathogenicity in mice is required for transcription of viral immediate-early genes in macrophages. Virology (1988) 163(2):397–404. 10.1016/0042-6822(88)90280-2 2833015

[35] Cook CH Trgovcich J Zimmerman PD Zhang Y Sedmak DD. Lipopolysaccharide, tumor necrosis factor alpha, or interleukin-1beta triggers reactivation of latent cytomegalovirus in immunocompetent mice. J Virol (2006) 80(18):9151–8. 10.1128/JVI.00216-06 16940526 PMC1563908

[36] Sanai N Alvarez-Buylla A Berger MS Neural stem cells and the origin of gliomas. N Engl J Med (2005) 353(8):811–22 10.1056/NEJMra043666 16120861

[37] Sinclair J Sissons P. Latency and reactivation of human cytomegalovirus. J Gen Virol (2006) 87(Pt 7):1763–79. 10.1099/vir.0.81891-0 16760381

[38] Price RL Song J Bingmer K Kim TH Yi JY Nowicki MO Cytomegalovirus contributes to glioblastoma in the context of tumor suppressor mutations. Cancer Res (2013) 73(11):3441–50. 10.1158/0008-5472 23729642 PMC4136413

[39] Kwon CH Zhao D Chen J Alcantara S Li Y Burns DK Pten haploinsufficiency accelerates formation of high grade astrocytomas. Cancer Res (2008) 68(9):3286–94. 10.1158/0008-5472.CAN-07-6867 18451155 PMC2760841

[40] Kim TH Song J Alcantara Llaguno SR Murnan E Liyanarachchi S Palanichamy K Suppression of peroxiredoxin 4 in glioblastoma cells increases apoptosis and reduces tumor growth. PLoS One (2012) 7(8):e42818. 10.1371/journal.pone.0042818 22916164 PMC3419743

[41] Jacks T Remington L Williams BO Schmitt EM Halachmi S Bronson RT Tumor spectrum analysis in p53-mutant mice. Curr Biol (1994) 4(1):1–7 10.1016/S0960-9822(00)00002-6 7922305

[42] Bongers G Maussang D Muniz LR Noriega VM Fraile-Ramos A Barker N The cytomegalovirus-encoded chemokine receptor US28 promotes intestinal neoplasia in transgenic mice. J Clin Invest (2010) 120(11):3969–78. 10.1172/JCI42563 20978345 PMC2964974

